# Functional Connectivity Changes in Multiple-Frequency Bands in Acute Basal Ganglia Ischemic Stroke Patients: A Machine Learning Approach

**DOI:** 10.1155/2022/1560748

**Published:** 2022-03-20

**Authors:** Jie Li, Lulu Cheng, Shijian Chen, Jian Zhang, Dongqiang Liu, Zhijian Liang, Huayun Li

**Affiliations:** ^1^Research Center of Brain and Cognitive Neuroscience, Liaoning Normal University, Dalian, China; ^2^Key Laboratory of Brain and Cognitive Neuroscience, Liaoning Province, China; ^3^School of Foreign Studies, China University of Petroleum (East China), Qingdao, China; ^4^Shanghai Center for Research in English Language Education, Shanghai International Studies University, Shanghai, China; ^5^Department of Neurology, The First Affiliated Hospital of Guangxi Medical University, Nanning, China; ^6^College of Teacher Education, Zhejiang Normal University, Jinhua, China; ^7^Key Laboratory of Intelligent Education Technology and Application, Zhejiang Normal University, Jinhua, China

## Abstract

**Purpose:**

Several functional magnetic resonance imaging (fMRI) studies have investigated the resting-state functional connectivity (rs-FC) changes in the primary motor cortex (M1) in patients with acute basal ganglia ischemic stroke (BGIS). However, the frequency-specific FC changes of M1 in acute BGIS patients are still unclear. Our study was aimed at exploring the altered FC of M1 in three frequency bands and the potential features as biomarkers for the identification by using a support vector machine (SVM).

**Methods:**

We included 28 acute BGIS patients and 42 healthy controls (HCs). Seed-based FC of two regions of interest (ROI, bilateral M1s) were calculated in conventional, slow-5, and slow-4 frequency bands. The abnormal voxel-wise FC values were defined as the features for SVM in different frequency bands.

**Results:**

In the ipsilesional M1, the acute BGIS patients exhibited decreased FC with the right lingual gyrus in the conventional and slow-4 frequency band. Besides, the acute BGIS patients showed increased FC with the right medial superior frontal gyrus (SFGmed) in the conventional and slow-5 frequency band and decreased FC with the left lingual gyrus in the slow-5 frequency band. In the contralesional M1, the BGIS patients showed lower FC with the right SFGmed in the conventional frequency band. The higher FC values with the right lingual gyrus and left SFGmed were detected in the slow-4 frequency band. In the slow-5 frequency band, the BGIS patients showed decreased FC with the left calcarine sulcus. SVM results showed that the combined features (slow-4+slow-5) had the highest accuracy in classification prediction of acute BGIS patients, with an area under curve (AUC) of 0.86.

**Conclusion:**

Acute BGIS patients had frequency-specific alterations in FC; SVM is a promising method for exploring these frequency-dependent FC alterations. The abnormal brain regions might be potential targets for future researchers in the rehabilitation and treatment of stroke patients.

## 1. Introduction

Stroke can be divided into two categories: ischemic and hemorrhagic cerebrovascular disease [1–3], and about 80% of stroke pertains to ischemic [4, 5]. Ischemic stroke is one of the most common diseases which may cause adult death or disability, in which acute basal ganglia ischemic stroke (BGIS) is attributed to the lesion of basal ganglia, and it is primarily associated with motor deficits in poststroke [6–8]. Motor function deficits are one of the main causes of disability in stroke patients and will seriously affect the patient's ability to live independently [9–11].

The primary motor cortex (M1) is one of the main brain regions involved in motor functions and the focus of many researchers exploring the neural mechanisms of motor function deficits. Resting-state functional magnetic resonance imaging (rs-fMRI) is a noninvasive neuroimaging tool with the advantage of no task demand or external stimulation and therefore demonstrated to be in the examination of brain functional deficits associated with a variety of neurological disorders [9, 10]. Functional connectivity (FC) is one of the most widely used methods in rs-fMRI [12–14], and it examined connectivity between two spatial regions of interest to quantify temporal coherence in rs-fMRI signal [15, 16]. Many studies have observed alterations of FC in stroke patients and showed that the abnormal connectivity related to behavioral deficits [17–21] and could provide crucial information on the neural mechanisms of motor recovery [22].

Up to now, FC was widely used for exploring the neurological mechanism of motor deficits in poststroke [23–25]. Studies showed that abnormal FC in stroke patients was correlated with their symptoms and deficits, and change in the strength of FC has also been found to be related to the change in the clinical assessment of motor function [26, 27]. For example, Zhang et al. found that patients with stroke had increased FC between the M1 and the inferior parietal cortex (IPL), frontal gyrus, supplementary motor area (SMA), and contralesional angular, while decreased FC was shown between the ipsilesional M1 and bilateral M1 [28]. Another study showed that poststroke subjects had an asymmetrical pattern of FC, which affected the hemisphere sensory cortex and was correlated with stroke severity [29]. However, previous FC studies mainly focused on the conventional band (0.01-0.08 Hz) [23–25, 30].

The human brain is a complex biological system that can generate a large number of oscillatory waves, and neural signals within different frequency bands exhibit different properties and physiological functions [31, 32]. Previous studies have subdivided the low-frequency range into four subfrequency bands [33, 34], among which gray matter activities mainly concentrated in the slow-4 (0.027-0.073 Hz) and slow-5 (0.01-0.027 Hz) frequency bands [34]. We examine the slow-5 and slow-4 bands because they have overlap with most of the conventional frequency bands and have minimal overlap with potential physiological noise frequency [34, 35]. Slow-4 has higher test-retest reliability and is more reliable for evaluating fMRI fluctuation amplitude signal than slow-5, and slow-5 and slow-4 showed higher power in different brain regions [34]. Moreover, recent studies have demonstrated that FC exhibited frequency-specific abnormalities in some neurological and psychiatric diseases and showed that fMRI signals in specific frequency bands might provide us with more sensitive information to understand the pathological mechanisms of disease [31, 32, 36]. Therefore, combining the importance of M1 brain regions for stroke patients, it is necessary to explore the frequency-specific alterations of FC in acute BGIS patients. In addition, to better understand disease detection sensitivity in frequency-specific FC, the machine learning approach is a powerful tool to classify patients from healthy controls. Support vector machine (SVM) was often used in MRI classification to detect biomarkers based on neuroimaging data, so that the contributing features in the classification model could help us have an in-depth understanding of the neurological mechanism of motor deficit in acute BGIS patients.

In this study, we used bilateral M1s as the regions of interest to determine whether stroke patients show abnormal FC between M1 and other voxels of the whole brain in three frequency bands (conventional, slow-5, and slow-4). Second, we explored the relationship between frequency-specific FC and clinical assessments. Third, we adopted SVM for classification with significantly different brain regions as imaging features. We hypothesized that FC changes in acute BGIS patients would exhibit frequency band specificity and that these abnormal FC could serve as effective biomarkers to identify patients and HCs, which might help us to better understand the neural mechanisms of motor deficit and provide support for future clinical rehabilitation treatment in acute BGIS patients.

## 2. Materials and Methods

### 2.1. Participants

We recruited 43 acute BGIS patients at the Department of Neurology, the First Affiliated Hospital of Guangxi Medical University. Patients were included using the following inclusion criteria: (1) patients were first onset acute BGIS (a consensus diagnosis was determined by a clinical neurologist and a radiologist); (2) age of patients was from 30 to 70 years; (3) the illness duration of BGIS patients ranged from 1 to 7 days (details shown in Table S1); (4) patients were right-handed before stroke; and (5) the National Institutes of Health Stroke Scale (NIHSS) scores of patients were no more than 8 (0 and 8 were included). Our study was approved by the Ethics Committee of the First Affiliated Hospital of Guangxi Medical University, and written informed consent was obtained from each participant. Besides, from the local community, we recruited 47 matched HCs who have no physical diseases or history of psychiatric or neurologic disorders.

To reduce the effect of confounding factors, we adopted a set of exclusion criteria: (1) inability to complete clinical scales, such as severe aphasia and auditory and/or visual disorder; (2) other neurological disorders which would affect the experiment, such as hemorrhage, multiple infarcts, leukoaraiosis, migraine, epilepsy, or psychiatric diseases; (3) any MRI contraindications; and (4) head motion exceeding 3 mm or 3°. Finally, our study included 28 acute BGIS patients and 42 HCs in the further analysis (2 BGIS patients and 1 HC had excessive head movement, the other 2 BGIS patients and 1 HC had incomplete data, and 11 BGIS patients and 3 HC were excluded for age and illness duration).

### 2.2. Clinical Scale Assessment

Patients were assessed by NIHSS to characterize the stroke severity and neurological deficits [37] and assessed by the Fugl-Meyer Assessment scale (FMA) to characterize motor impairment [38]. Patients were assessed by NIHSS to characterize the stroke severity and neurological deficits [37] and assessed by the Fugl-Meyer Assessment scale (FMA) to characterize motor impairment [38].

### 2.3. Image Acquisition

Images were acquired using a 3.0 T Siemens Prisma MRI scanner with a 64-channel phased array head coil. The imaging parameters of resting-state fMRI data were as follows: repetition time (TR) = 2000 ms, echo time (TE) = 35 ms, voxel size = 2.6 × 2.6 × 3 mm^3^, matrix size = 64 × 64, field of view (FOV) = 240 × 240 mm^2^, flip angle (FA) = 90°, slice number = 40, 6 minutes and 12 seconds, and 186 volumes. The acquisition parameters for anatomical T1-weighted images were as follows: TR = 2300 ms, TE = 2.98 ms, voxel size = 1 × 1 × 1 mm^3^, matrix size = 256 × 256, FOV = 256 × 256 mm^2^, FA = 9°, slice number = 176, and 5 minutes and 21 seconds. All participants were required to remain awake, close their eyes, and keep relaxing during scanning.

### 2.4. Image Preprocessing

All images were preprocessed by using Statistical Parametric Mapping (SPM 12, http://www.fil.ion.ucl.ac.uk/spm) and Resting-State fMRI Data Analysis Toolkit plus (RESTplus V1.24, http://restfmri.net/forum/restplus) [39], implemented in the MATLAB R2017b platform (https://ww2.mathworks.cn/products/matlab.html). Briefly, the preprocessing steps included the following steps. (1) Remove the first ten volumes of each functional image and then keep 176 volumes. (2) Slice-time correction. (3) Realignment. (4) Normalization. The realigned images were spatially normalized to the Montreal Neurological Institute (MNI) space and resampled with a voxel size of 3 × 3 × 3 mm^3^. (5) Spatial smoothing with 6 mm full width at half maximum (FWHM). (6) Nuisance covariate regression. The nuisance regressors included the Friston-24 motion parameters [40], white matter signals [41], cerebrospinal fluid signals [41], and global mean signals [42]. We also removed the linear trends. (7) Filtering. The temporal band-pass filtering was, respectively, performed in the three frequency bands: conventional (0.01-0.08 Hz), slow-5 (0.01–0.027 Hz), and slow-4 (0.027–0.073 Hz).

### 2.5. Functional Connectivity Analyses

To calculate seed-based functional connectivity, we defined two regions of interest (ROIs) of the M1 with 6 mm diameter spheres. The ROIs are the left primary motor cortex (M1_L, MNI coordinates, -12, -30, 54) and the right primary motor cortex (M1_R, MNI coordinates, 12, -30, 54) in line with a previous study [43]. In three frequency bands, we then computed Pearson correlation coefficients between each ROI and the voxels of the whole brain to create correlation FC maps. Finally, we converted the correlation maps into *Z* values using Fisher's *r*-to-*Z* transformation to improve normality. Notably, the lesion side in the right basal ganglia of 15 cases was flipped to the left by the RESTplus V1.25 toolbox based on the MATLAB R2017b platform. Thus, we defined the M1_L as the ipsilesional M1 and defined the M1_R as the contralesional M1.

### 2.6. Statistical Analyses

All statistical analyses were conducted by the Statistical Product and Service Solutions version (SPSS 26.0, IBM, Armonk, NY, USA). The age and education of participants were compared using two-sample *t*-tests, and the gender of participants was compared using chi-squared tests (significance level: *p* < 0.05).

Two-sample *t*-tests were performed to identify the FC differences between acute BGIS patients and HCs in three frequency bands. We also applied the Gaussian random field (GRF) theory multiple comparison correction (voxel-level: *p* < 0.005, cluster-level: *p* < 0.05).

### 2.7. Correlation Analyses

To explore the relationship between the FC abnormalities and the function impairments, we then performed the Pearson correlation analysis between the aberrant FC and clinical scales (NIHSS and FMA). The statistical significance level was set at *p* < 0.05.

### 2.8. Feature Extraction and SVM Analyses

Abnormal brain regions were obtained from the group comparisons between patients and controls. The intergroup *z* values of FC difference were used as the classification features in this study. Specifically, we extracted voxel-wise *z* FC values from these brain clusters in each ROI in three frequency bands. Besides, *z* values of FC from abnormal brain regions in the conventional and subfrequency band (slow-4+slow-5) were also extracted and combined as features.

In this study, we employed the LIBSVM software package in MATLAB, in which a linear kernel support vector machine was conducted within each cluster in three frequency bands (http://www.csie.ntu.edu.tw/~cjlin/libsvm/) [44]. To reduce the risk of overfitting the training data, the SVM with linear kernel was used to extract the feature weights directly [45]. In the SVM model, the parameter *C* was set to 1, and the optimized linear kernel parameter *γ* was set to 2^*N*^ (*N* ranges from −5 to 5). The performance of the SVM classifier was evaluated by a leave-one-out cross-validation method (LOOCV). The data of one participant is selected as the test sample, and data of other participants were used to train the SVM classifier. To evaluate the overall accuracy of the SVM, we repeated the classification test for each pair of participants. The performance of classification was evaluated by calculating the accuracy, sensitivity, specificity, precision, and area under the receiver operating characteristic (ROC) curve (AUC) [46].

## 3. Results

### 3.1. Demographic and Clinical Characteristics of the Participants

Our study included 70 subjects. Demographic and clinical data of all participants are summarized in Table 1. There was no significant difference between acute BGIS patients and HCs in age and education (*p* > 0.05). The significant difference between the two groups was found in gender (*p* < 0.0001).

### 3.2. Functional Connectivity Alterations of the Ipsilesional M1

**Figure 1:**
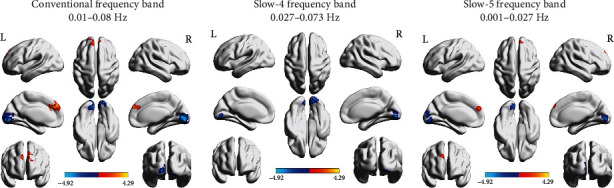
The functional connectivity alterations of the ipsilesional M1 in the three frequency bands.

In the conventional frequency band (0.01-0.08 Hz), the acute BGIS patients showed decreased FC values between the ipsilesional M1 and the right lingual gyrus and increased FC values between the ipsilesional M1 and the right medial superior frontal gyrus (SFGmed) compared with the HCs. In the slow-4 frequency band (0.027-0.073 Hz), acute BGIS patients exhibited decreased FC values between the ipsilesional M1 and the right lingual gyrus. In addition, we also found the decreased FC values between the ipsilesional M1 and the left lingual gyrus and the increased FC values between the ipsilesional M1 and the right SFGmed in the slow-5 frequency band (0.01-0.027 Hz) (details shown in Figure 1 and Table 2).

### 3.3. Functional Connectivity Alterations of the Contralesional M1

**Figure 2:**
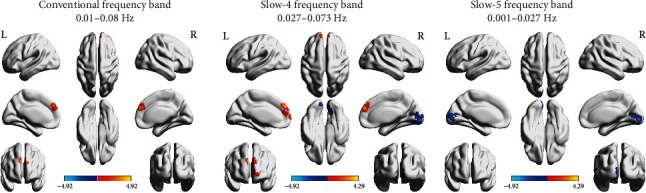
The functional connectivity alterations of the contralesional M1 in the three frequency bands.

In the conventional frequency band, results showed that acute BGIS patients had increased FC values between the contralesional M1 and the right SFGmed. As for the slow-4 band, acute BGIS patients exhibited higher FC values in the left SFGmed and lower FC values in the right lingual gyrus. In the slow-5 frequency band, we found that acute BGIS patients had increased FC values between the contralesional M1 and the left calcarine sulcus (shown in Figure 2 and Table 3).

### 3.4. The Relationship between Abnormal FC Values and Clinical Scores

We performed the Pearson correlation analysis between FC values and clinical scores (including NIHSS and FMA scores) in all three frequency bands. No significant correlation was found between abnormal FC values and clinical scores (*p* < 0.05).

### 3.5. Classification Results

**Figure 3:**
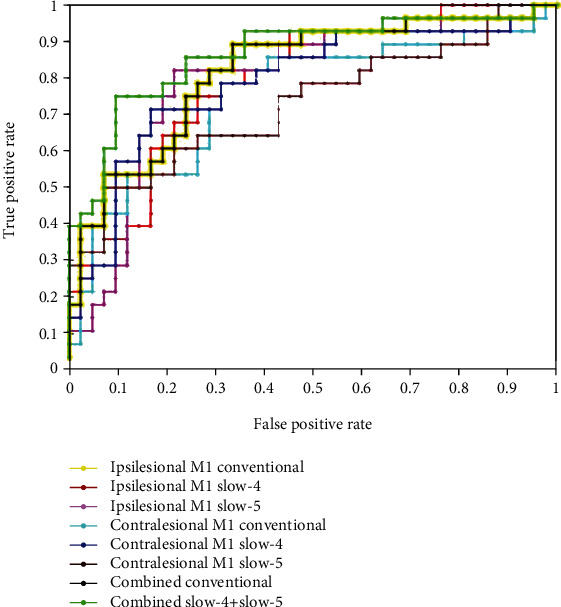
The receiver operating characteristic (ROC) curve in different features. The yellow line with a dot represents the ROC curve of the features in ipsilesional M1 in the conventional frequency band. The red line with a dot represents the ROC curve of the features in ipsilesional M1 in slow-4. The pink line with a dot represents the ROC curve of the features in ipsilesional M1 in slow-5. The cyan line with a dot represents the ROC curve of the features in contralesional M1 in the conventional frequency band. The blue line with a dot represents the ROC curve of the features in contralesional M1in slow-4. The brown line with a dot represents the ROC curve of the features in contralesional M1 in slow-5. The black line with a dot represents the ROC curve of the combined features in the conventional frequency band. The green line with a dot represents the ROC curve of the combined features in subfrequency bands (slow-4+slow-5).

In the current study, Table 4 summarizes and shows the accuracies, sensitivities, specificities, and precisions of the classification for features in each ROI in three frequency bands and the combined features. The receiver operating characteristic (ROC) curve of the classifier for each feature is shown in Figure 3. Features in each of the three frequency bands and combined features demonstrated a significantly higher accuracy rate and AUC value than chance (*p* < 0.05). Specifically, both single features and the combined features had AUC values up to 0.73. In bilateral M1s, features in the conventional band had close AUC values ranging from 0.75 to 0.82. As for subbands (slow-4 and slow-5), the result also showed similar AUC values. The combined features in the conventional frequency band had the same classification performance AUC values of 0.82. Moreover, the combined features in subfrequency bands (slow-4 and slow-5) had the highest accuracy (80.00%) with an AUC value of 0.86.

## 4. Discussion

In our study, we explored the FC changes of the bilateral M1s in acute BGIS patients at three frequency bands, and we also adopted a machine learning approach based on SVM to identify the valuable neuroimaging biomarkers classifying the acute BGIS patients and HCs. The main findings of our study are the following. (1) In the conventional frequency band (0.01-0.08 Hz), acute BGIS patients showed significantly altered FC between the ipsilesional M1 and right media superior frontal gyrus (SFGmed) and right lingual gyrus, acute BGIS patients also showed increased FC values between contralesional M1 and the right SFGmed. (2) Changes of FC in patients with acute BGIS showed frequency bands specificity. (3) SVM results showed that features extracted based on the differences in subfrequency bands have the best performance in classification. Our results indicated that the altered FC in acute BGIS patients was frequency-dependent, and frequency-specific FC might provide us with new insights into the neural mechanisms in acute BGIS patients.

The M1 is not only involved in the motor performance and execution but also the planning, preparation, and learning process of the motor [47]. Besides, the M1 is also related to the recovery of motor function in stroke patients [48, 49]. We found significantly increased FC of bilateral M1s with right SFGmed in patients with acute BGIS, which further confirmed the previous conclusions that patients with stroke showed abnormal FC patterns in M1 with other brain regions [22, 50–52]. A previous study similarly found that stroke patients showed significantly increased FC between the ipsilesional M1 and the SFGmed and indicated that the FC changes were associated with motor function changes [28]. In addition, we also found decreased FC between ipsilesional M1 and right lingual gyrus in the acute BGIS patients. Similar to this, Stinear et al. showed that the excitability of ipsilesional M1 was reduced in acute stroke by using transcranial magnetic stimulation (TMS) [53]. Besides, previous evidence using task fMRI showed that efficient long-term motor recovery in stroke patients within the M1 area was related to reorganization within the surrounding motor cortex [54]. Our results further supported the findings that poststroke patients showed decreased local synchronization in the lingual gyrus and the right medial SFG, and the abnormal local synchronization had a positive correlation with aphasia severity [55]. In summary, the results indicated that the changes of FC between bilateral M1s and right SFGmed and the lingual gyrus might be related to the motor impairment of patients with BGIS.

In subfrequency bands, the FC of bilateral M1 showed frequency band specificity. As for ipsilesional M1, the FC of ipsilesional M1 with right lingual gyrus was more sensitive to the slow-4 than slow-5, whereas the FC of ipsilesional M1 with the right medial SFG and the left lingual gyrus were more sensitive to the slow-5 than slow-4. In terms of the contralesional M1, in the slow-5 band, the left calcarine sulcus showed decreased FC with contralesional M1 between acute BGIS patients and HCs. Hence, the FC of contralesional M1 with left media SFG and right lingual gyrus were more sensitive than the conventional band and the slow-5 band. Our findings were consistent with previous results, which showed that patients with stroke showed frequency-specific pathological characteristics. Zhu et al. demonstrated that abnormal changes in the regional properties of brain activity that occurred in the slow-5 band were more sensitive to the changes in the slow-4 [56]. Besides, the aberrant patterns of local synchronization were different in the slow-4 band and the slow-5 band in stroke patients [57]. These findings suggested that the FC patterns were sensitive to the specific frequency. Many studies have revealed frequency-dependent abnormalities in neurological and psychiatry diseases including autism spectrum disorder [58], schizophrenia [59, 60], and depression [36, 61]. Yu et al. demonstrated that schizophrenia patients showed larger local synchronization in the slow-4 band than in the slow-5 band in the fusiform gyrus, and superior frontal gyrus, whereas larger local synchronization in slow-5 was found in the culmen, parahippocampal gyrus, putamen, and dorsal middle prefrontal gyrus [60]. Besides, a recent study found that patients with bipolar disorder depression had increased functional interactions in the left pre-/postcentral gyrus, left fusiform gyrus (FG), and the left lingual gyrus (LG) in the slow-4 band, whereas patients showed decreased functional interactions in the left LG in the slow-5 band [36]. Previous studies demonstrated that the frequency specificities were widely presented in BOLD fMRI [62, 63]. Moreover, fMRI and electrophysiological studies have demonstrated that brain activities in independent frequency bands are related to specific properties and physiological functions [64, 65]. Therefore, we highlighted the importance of FC within different frequency bands in patients with acute BGIS.

SVM has been widely used as a diagnostic and predictive aid in the field of clinical disease. SVM results showed that both single feature and combined features in the conventional band had the same classification performance. However, the combined features in subfrequency bands (slow-4+slow-5) had the highest accuracy (80.00%) with an AUC value of 0.86. It indicated that FC changes in specific frequency bands could provide us with more sensitive information than the conventional frequency band. SVM results demonstrated abnormal FC values in bilateral M1s with the lingual gyrus and the media SFG in different frequency bands, which could be used to distinguish patients with acute BGIS from HCs with satisfactory accuracy, specificity, and sensitivity and precision, and facilitate the establishment of diagnostic indicators. Therefore, we inferred that abnormal FC values in subfrequency bands (slow-4+slow-5) could be used as a potential imaging biomarker to differentiate patients from controls. Previous findings have also revealed fMRI signal in the subfrequency band had a good diagnostic potential for Parkinson's disease and anxiety disorder [66, 67].

Moreover, many studies predefined the bilateral M1 as the target stimulus location of transcranial magnetic stimulation (TMS), which generates a descending volley in the corticospinal pathway, and elicits a motor evoked potential (MEP) in muscles of the contralateral limb [68]. Studies in acute stroke patients indicated that TMS on motor regions can lead to improvements in motor function [68]. One recent study explored frequency-dependent stimulation effects in a combined rTMS–fMRI approach and found that changes in FC strength because of low-frequency rTMS were even detectable 7 days after stimulation [69]. Combined with this result, future researchers might combine TMS technology and frequency-specific FC changes in M1 to help treat and recover for motor deficits in stroke patients. The exploration of the neural mechanisms of motor deficit in acute BGIS patients is necessary and might provide support for future clinical rehabilitation treatment in acute BGIS patients.

Taken together, the current results indicated that patients with acute BGIS showed frequency-specific FC abnormalities, which provided valuable information and neuroimaging biomarkers for exploring the pathological mechanisms of acute BGIS patients.

## 5. Limitations

Firstly, our study had a relatively small sample size. Therefore, future researchers can explore the changes in the specificity of different frequency bands in a larger sample of patients with acute BGIS. Second, our study focused on the cross-sectional changes between the acute BGIS patients and HCs; future investigators should consider longitudinal frequency-specific changes in the acute BGIS patients.

## 6. Conclusion

Alterations of FC in acute BGIS patients are frequency-specific, and the specific information can be well detected by the machine learning approach. Our study might provide new insights into the pathophysiology of acute BGIS patients. The frequency-specific FC abnormal brain areas might provide inspiration and assistance for clinical treatment and rehabilitation of stroke patients.

## Figures and Tables

**Table 1:** Demographic and clinical information of participants.

	BGIS patients (*N* = 28)	HCs (*N* = 42)	*p* value
Age (years)	54.60 ± 8.90	54.85 ± 10.24	0.9
Gender (male/female)	23/5	19/23	0.0001
Education (years)	12.00 ± 3.23	12.28 ± 2.99	0.7
NIHSS score	3.60 ± 2.22	—	—
FMA score	77.03 ± 17.22	—	—

BGIS: basal ganglia ischemic stroke; HCs: healthy controls; NIHSS: National Institutes of Health Stroke Scale; FMA: Fugl-Meyer Assessment.

**Table 2:** The functional connectivity alterations of the ipsilesional M1 in the three frequency bands.

Brain regions (BGIS patients > HCs)	Number of voxels	MNI coordinate			Peak *t* value
		*x*	*y*	*z*	
Conventional frequency band (0.01-0.08 Hz)					
Lingual_R	469	3	-84	-9	-4.9214
Frontal_Sup_Medial_R	171	15	57	42	4.2933
Slow-4 frequency band (0.027-0.073 Hz)					
Lingual_R	168	3	-84	-9	-4.6060
Slow-5 frequency band (0.01-0.027 Hz)					
Lingual_L	132	-3	-81	-3	-3.8903
Frontal_Sup_Medial_R	80	15	57	42	4.2933

Lingual_R: right lingual gyrus; Lingual_L: left lingual gyrus; Frontal_Sup_Medial_R: right medial superior frontal gyrus; MNI: Montreal Neurological Institute.

**Table 3:** The functional connectivity alterations of the contralesional M1 in the three frequency bands.

Brain regions (BGIS patients > HCs)	Number of voxels	MNI coordinate			Peak *t* value
		*x*	*y*	*z*	
Conventional frequency band (0.01-0.08 Hz)					
Frontal_Sup_Medial_R	128	6	54	30	3.9253
Slow-4 frequency band (0.027-0.073 Hz)					
Lingual_R	179	12	-76	-12	-4.733
Frontal_Sup_Medial_L	216	0	48	24	4.6441
Slow-5 frequency band (0.01-0.027 Hz)					
Calcarine_L	137	-3	-96	6	-4.4748

Note: clusters located in the cerebellum are not reported. Lingual_R: right lingual gyrus; Frontal_Sup_Medial_R: right medial superior frontal gyrus; Frontal_Sup_Medial_L: left medial superior frontal gyrus; Calcarine_L: left calcarine sulcus; MNI: Montreal Neurological Institute.

**Table 4:** The results of a single or combined features in SVM Classification.

Feature	AUC	Accuracy (%)	Sensitivity (%)	Specificity (%)	Precision (%)
Ipsilesional M1					
Conventional	0.82	72.86	57.14	83.33	69.57
Slow-4	0.80	72.86	67.86	76.19	65.52
Slow-5	0.80	77.14	64.29	85.71	75.00
Contralesional M1					
Conventional	0.75	71.43	39.29	92.86	78.57
Slow-4	0.79	72.86	46.43	90.48	76.47
Slow-5	0.73	68.57	64.29	71.43	60.00
Combined					
Conventional	0.82	72.86	57.14	83.33	69.57
Slow-4+Slow-5	0.86	80.00	60.71	92.86	85.00

## Data Availability

The raw data supporting the conclusions of this article will be made available by the authors, without undue reservation.

## Supplementary Materials

Supplementary Materials Table S1: The time of functional magnetic resonance scanning after stroke.
