# Supplementary material for: Functional Connectivity Changes in Multiple-Frequency Bands in Acute Basal Ganglia Ischemic Stroke Patients: A Machine Learning Approach
Source: Neural Plast. 2022 Mar 20;2022:1560748. doi: 10.1155/2022/1560748 (PMC8958111; doi:10.1155/2022/1560748)
Supplement: Supplementary Materials — Table S1: The time of functional magnetic resonance scanning after stroke. [file 1560748.f1.docx]

| **Number of subjects** | **Duration (days)** | **Number of subjects** | **Duration (days)** |
| --- | --- | --- | --- |
| patient001 | 4 | patient015 | 4 |
| patient002 | 6 | patient016 | 4 |
| patient003 | 2 | patient017 | 6 |
| patient004 | 7 | patient018 | 6 |
| patient005 | 7 | patient019 | 1 |
| patient006 | 1 | patient020 | 6 |
| patient007 | 5 | patient021 | 4 |
| patient008 | 7 | patient022 | 2 |
| patient009 | 2 | patient023 | 3 |
| patient010 | 1 | patient024 | 3 |
| patient011 | 1 | patient025 | 1 |
| patient012 | 1 | patient026 | 4 |
| patient013 | 2 | patient027 | 3 |
| patient014 | 3 | patient028 | 1 |

**Supplementary materials**

**Table S1** The time of functional magnetic resonance scanning after stroke
